# Estimation of the flow rate of pyrolysis gasoline, ethylene, and propylene in an industrial olefin plant using machine learning approaches

**DOI:** 10.1038/s41598-023-41273-4

**Published:** 2023-08-28

**Authors:** Jafar Abdi, Golshan Mazloom, Fahimeh Hadavimoghaddam, Abdolhossein Hemmati-Sarapardeh, Seyyed Hamid Esmaeili-Faraj, Akbar Bolhasani, Soroush Karamian, Shahin Hosseini

**Affiliations:** 1 Faculty of Chemical and Materials Engineering, Shahrood University of Technology, Shahrood, Iran; 2 Department of Chemical Engineering, Faculty of Engineering, University of Mazandaran, Babolsar, Iran; 3 Institute of Unconventional Oil & Gas, Northeast Petroleum University, Daqing, 163318 Heilongjiang China; 4 Ufa State Petroleum Technological University, Ufa, 450064 Russia; 5 Department of Petroleum Engineering, Shahid Bahonar University of Kerman, Kerman, Iran; 6 State Key Laboratory of Petroleum Resources and Prospecting, China University of Petroleum (Beijing), Beijing, China; 7 Research and Development Center, Jam Petrochemical Company, Bushehr, 1434853114 Iran

**Keywords:** Energy science and technology, Engineering

## Abstract

Light olefins, as the backbone of the chemical and petrochemical industries, are produced mainly via steam cracking route. Prediction the of effects of operating variables on the product yield distribution through the mechanistic approaches is complex and requires long time. While increasing in the industrial automation and the availability of the high throughput data, the machine learning approaches have gained much attention due to the simplicity and less required computational efforts. In this study, the potential capability of four powerful machine learning models, i.e., Multilayer perceptron (MLP) neural network, adaptive boosting-support vector regression (AdaBoost-SVR), recurrent neural network (RNN), and deep belief network (DBN) was investigated to predict the product distribution of an olefin plant in industrial scale. In this regard, an extensive data set including 1184 actual data points were gathered during four successive years under various practical conditions. 24 varying independent parameters, including flow rates of different feedstock, numbers of active furnaces, and coil outlet temperatures, were chosen as the input variables of the models and the outputs were the flow rates of the main products, i.e., pyrolysis gasoline, ethylene, and propylene. The accuracy of the models was assessed by different statistical techniques. Based on the obtained results, the RNN model accurately predicted the main product flow rates with average absolute percent relative error (AAPRE) and determination coefficient (R^2^) values of 1.94% and 0.97, 1.29% and 0.99, 0.70% and 0.99 for pyrolysis gasoline, propylene, and ethylene, respectively. The influence of the various parameters on the products flow rate (estimated by the RNN model) was studied by the relevancy factor calculation. Accordingly, the number of furnaces in service and the flow rates of some feedstock had more positive impacts on the outputs. In addition, the effects of different operating conditions on the propylene/ethylene (P/E) ratio as a cracking severity factor were also discussed. This research proved that intelligent approaches, despite being simple and straightforward, can predict complex unit performance. Thus, they can be efficiently utilized to control and optimize different industrial-scale units.

## Introduction

Light olefins, i.e., propylene and ethylene, are among the most valuable petrochemicals^1, 2^, primarily used as building blocks of different polymers, plastics, resins, fibers, elastomers, etc.^3^. Due to their wide range of applications in industries, their global marketing would continue to grow. There are different alternative technologies for light olefin production depending on the feedstock type^1, 2^. The conventional technology, widely used in industries, is steam cracking of hydrocarbons^4^. The feedstock can be varied from gaseous, including butane, ethane, propane, and liquid feeds such as naphtha, liquid petroleum gas, Gasoil, and also methanol^5^. The olefin units can be divided into complicated subsections; furnaces, quench subsections, compression subsections, and separation columns. To approach the highest yield of propylene and ethylene, different parameters, including feedstock mixture, sections configuration, and operating conditions, have to be controlled and optimized. Developing an efficient model is a convenient tool for investigating the influences of different variables and predicting the product yield^6–8^. The modeling and simulation of olefin units have been studied significantly by various researchers^9–12^. For example, Zargani et al.^9^ have studied the performance of HZSM-5 zeolite for the methanol-to-olefins process in a honeycomb monolith reactor and a fixed bed reactor containing cylindrical shaped catalyst by two-dimensional two-scale heterogeneous models. The effects of space–time, feed water content, and monolith cell density on the temperature distribution, conversions, and olefin selectivity are investigated. Their modeling results indicated that the conversion, selectivity, and olefin yields in the monolithic reactor are higher than those of the fixed bed reactor. Zhang et al.^10^ have simulated an industrial steam cracking unit with floor burners using the computational fluid dynamics (CFD) approach and the effects of the flue gas radiative properties and the burner geometry were evaluated on the heat transfer. Two models were implemented; the nongray gas radiative model (NGGRM) and the gray gas radiative model (GGRM). Their obtained results revealed that NGGRM provides more acceptable results for the thermal efficiency and the flue gas outlet temperature as compared with the industrial data^10, 11^. Recently, Fakhroleslam and Sadrameli have critically reviewed different approaches for mathematical modeling and simulation of olefin plants such as mechanism and kinetic modeling, rigorous modeling, abstract modeling and CFD simulation^12^.

Since the olefin units include different complicated operations, their modeling using mechanistic models is CPU-consuming and needs a lot of time and attempts. In this regard, other reliable and accurate approaches should be developed. Different machine learning (ML) models are among these alternative methods that are developed for modeling these aforementioned complex processes. In recent years, ML methods have attracted much attention in catalyst design, kinetic modeling, reactor modeling, and process conditions^13–20^. Azarhoosh et al.^21^ evaluated artificial neural networks (ANN) and genetic programming (GP) for studying the performance of SAPO-34 in the methanol-to-olefin process. The influence of different parameters on catalyst preparation, including ultrasonic intensity, the time of crystallization and irradiation, carbon nanotube, and organic template contents, on the products selectivity was modeled. Sedighi et al.^22^ have developed kinetic, ANN, neuro-fuzzy (NF), and polynomial models to study the effects of furnace temperature, steam content, and flow rate of the feed on the products yield in the cracking of liquid hydrocarbons to olefins. Different ML models based on the ANN were established by Zhu et al.^23^ to discuss the effects of temperature and catalyst in n-heptane cracking on propylene and ethylene. Peng et al.^24^ have developed an intelligent model based on the artificial bee colony (ABC) and NF to predict cooking time factor in cracking furnaces.

In this study, some ML models are implemented for simulating a real industrial-scale olefin plant with the whole of the subsections. The real experimental data are collected during four successive years from the olefin unit of Jam petrochemical company under the various conditions of different flow rates of feedstock, numbers of active gas and liquid furnaces, and coil outlet temperature. Then, four powerful ML models, including adaptive boosting-support vector regression (AdaBoost-SVR), Multi-layer perceptron (MLP) neural network, deep belief network (DBN), and recurrent neural network (RNN), are implemented for modeling the main products flow rates, i.e., ethylene, propylene, and pyrolysis gasoline (PYG). The influence of the various parameters on the products flow rate is studied by the relevancy factor calculation. In addition, the effects of different operating conditions on the propylene/ethylene (P/E) ratio as a cracking severity factor are also discussed.

## The olefin plant description

**Figure 1:**
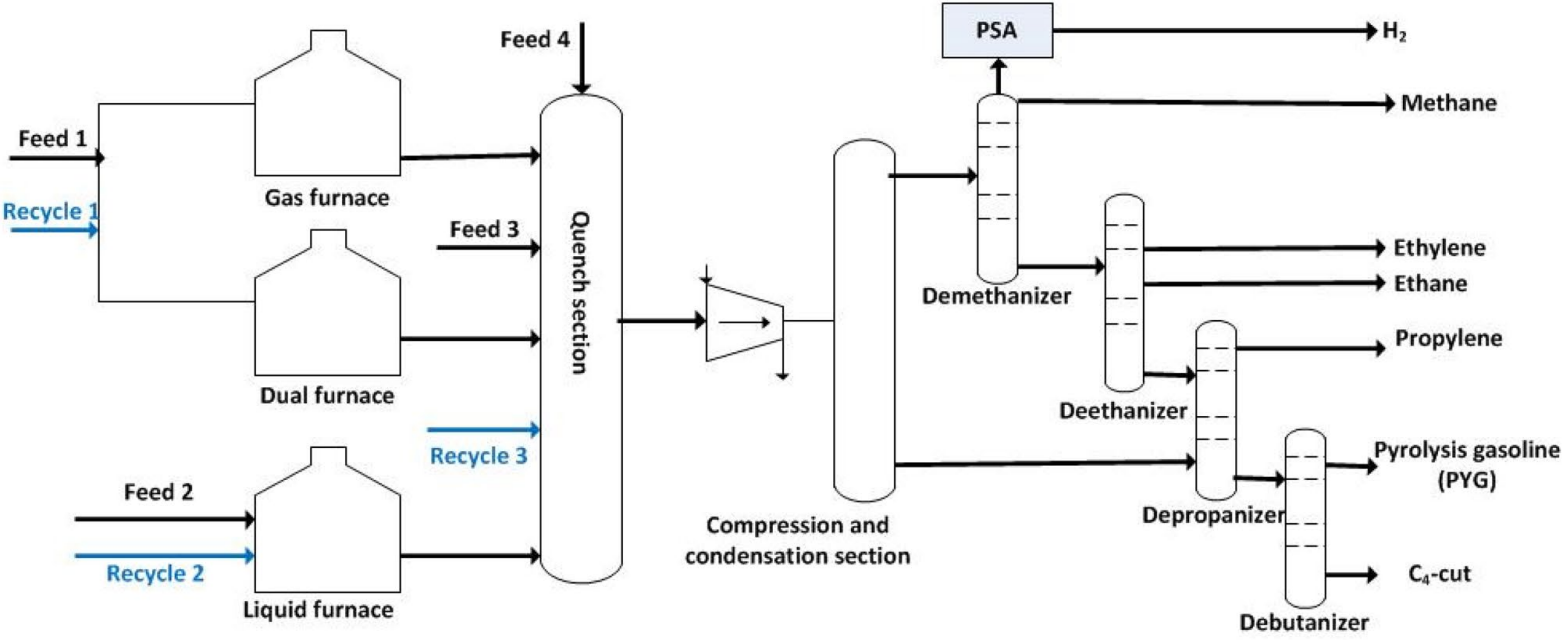
Schematic representation of different sections of the olefin plant of JPC.

The simulation was carried out for the olefin plant in Jam petrochemical company (JPC) located in Pars special economic energy zone as an industrial case study. Different sections of the olefin plant were briefly described and schematically presented in Fig. 1. At the start of the olefin plant, the section of furnaces is located. Based on feedstock type, the furnaces are classified into liquid, gas, and dual furnaces. The feedstock comprises four streams provided from battery limit (BL) and the other three streams recycled from down streams. At the end of thermal cracking, the product is directed to the quench tower, where the cracked gas is cooled rapidly to stop the undesired coke formation reactions. The recovery of different products is achieved by condensation and then demethanization process. The stream from the top of the de-methanizer flows to the pressure swing adsorption unit, where an H_2_ stream with ultra-high purity is produced. The flow from the bottom of the demethanizer is transferred to the de-ethanizer section to separate ethane and ethylene. C_3_ and C_4_ cut fractionation is continuously carried out in the de-propanizer and de-butanizer, respectively. The final products of the olefin unit are listed as ethylene, propylene, and PYG. However, different intermediates can be produced due to the complex reaction networks.

## The theory of ML models

### Multilayer perceptron (MLP) neural network

MLP is one of the most frequently used artificial intelligent approaches developed in the 1980s. A typical MLP model is composed of different layers; one input layer, one or more hidden layers, and at the end, one output layer^25^. The hidden layer(s) are responsible for establishing the connection between input and output layers using different transfer functions^26^. Table 1 lists different transfer functions widely used in different layers with their mathematical equations. Tangent sigmoid (*tansig*) and log-sigmoid (*logsig*) transfer functions are normally employed for hidden layers whenever the *purelin*, as a linear function, is employed for the output layer. In MLP, each layer possesses a determined number of neurons. The number of input layer neurons is equal to the input parameters. However, the number of hidden layers and the number of neurons in each hidden layer are tuned to estimate the experimental data with the best accuracy^27^. While the output layer typically includes only one neuron. The neurons of each layer are connected by tunable parameters named biases and weights. The relationship among input and output parameters is determined using training of the network, which is a process for determining weights and biases to minimize the errors between model-predicted and real experimental values^28^. Different optimization methods such as Levenberg–Marquardt (LM) can be used in network training. The output value of an MLP model with two hidden layers is calculated based on Eq. (1), in which tansig and logsig are transfer functions of the first and second hidden layers, respectively, and purelin belongs to the output layer^26^.

1 $$Output=purelin\left({w}_{3}\times \left(logsig\left({w}_{2}\times \left(tansig\left({w}_{1}\times x\right)+{b}_{1}\right)\right)+{b}_{2}\right)\right)+{b}_{3}$$

where w, b, and x denote weights, biases, and input data vector. Logsig, tansig, and purelin are activation functions.

**Table 1:** Frequently used transfer functions.

Transfer function	Mathematical expression
tansig or tanh	$f\left(x\right)= \frac{{e}^{x}-{e}^{-x}}{{e}^{x}+{e}^{-x}}=\frac{2}{1-{e}^{-2x}}-1$
logsig or sigmoid	$f\left(x\right)=\frac{1}{1+{e}^{-x}}$
Sinusid	$f\left(x\right)=\mathrm{sin}(x)$
Purelin	$f\left(x\right)=x$
Arctan	$f\left(x\right)={tan}^{-1}(x)$

### Adaptive boosting (AdaBoost)

**Figure 2:**
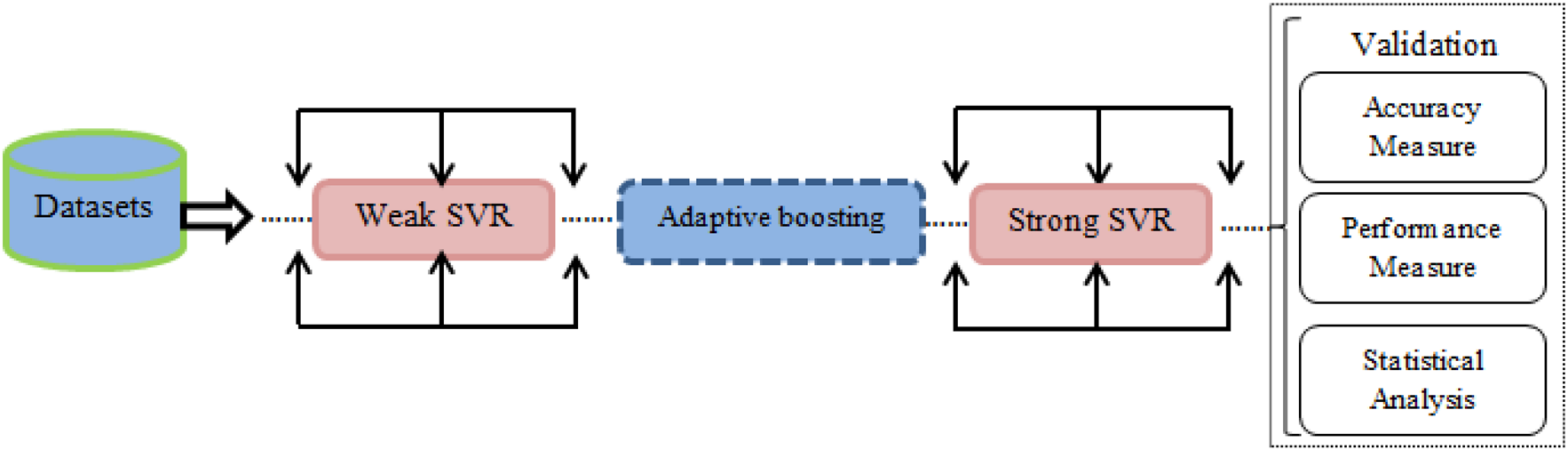
Schematically representation of the developed Adaboost model.

Freund and Schapire^29, 30^ invented AdaBoost, a reasonably popular approach. The AdaBoost approach is based on gradually integrating multiple weak classifiers, learning from each other's flaws to create a more accurate model. The AdaBoost method's basic phases are shown in Fig. 2.

The theory of the AdaBoost model is described as follows^29^:

- Given a weight to every data point. All data sets have the same weighted sample in the first step: ${w}_{j}=\frac{1}{n}, j=\mathrm{1,2},\dots .,n$, where n represents the number of training data.
- Using weights to provide training data set to the weak learner ${Wl}_{i}(x)$ and computing the weighted error:
  2 $${Err}_{i}=\frac{{\sum }_{j=1}^{n}{w}_{j}I({t}_{j}\ne {wl}_{i}\left(x\right))}{{\sum }_{j=1}^{n}{w}_{j}}, \quad I\left(x\right)=\{\begin{array}{l}0 \quad if\,\, x=false \\ 1 \quad if \,\, x=true\end{array}$$

That i is (1 to N), and N declares the total number of weak learners.

- From 1 to N, define the weights for all weak learners by Eq. (3):
  3 $${\beta }_{i}=log\left(\frac{\left(1-{Err}_{i}\right)}{{Err}_{i}}\right)$$
- Changing all of the weak learner's dataset weights from 1 to N.
- Assigning a weak learner to the data test (x) as a result.

The support vector regression (SVR) was the weak learner in this work's AdaBoost system (Fig. 2).

### Support vector machine (SVM)

Support vector machine (SVM) is a type of ML model that may be used for clustering (SVC) and regression (SVR)^31^. SVR has already received a lot of interest because of its ability to represent complicated structures. The basic principle of the SVR model is concisely given in this paper^31, 32^.

In the case of a data set $\left({x}_{i},{y}_{i}\right)$ in which $x\in {R}_{d}$ is the d-dimensional input data and $y\in R$ becomes an output vector, and the SVR model gives a function *f(x)* for each data set to estimate the output value as Eq. (4)^20^.

4 $$f\left(x\right)=w.\phi \left({x}_{i}\right)+b$$

Weight vector, bias, and the non-linear function are denoted by *w*, *b,* and $\phi \left(x\right)$, accordingly. The $\phi \left(x\right)$ function maps x from low dimensional space into n-dimensional feature space. Vapnik created an improvement approach to achieve the good values of the w and b vectors^33, 34^.

5 $$minimize \frac{1}{2}{w}^{T}w+C\sum_{j=1}^{N}\left({\upzeta }_{j}^{-}+{\zeta }_{j}^{+}\right)$$

6 $$\left\{\begin{array}{c}\left(w.\phi \left({x}_{i}\right)+b\right)-{y}_{i}\le \varepsilon +{\zeta }_{j}^{-}\\ {y}_{i}-\left(w.\phi \left({x}_{i}\right)+b\right)\le \varepsilon +{\zeta }_{j}^{+}\\ {\zeta }_{j}^{+},{\zeta }_{j}^{-}\ge 0,\quad i=\mathrm{1,2},\dots m\end{array}\right.$$

${\upzeta }_{\mathrm{j}}^{+}$ and ${\upzeta }_{\mathrm{j}}^{-}$ indicate the lower and higher excess variations, correspondingly. C is a regularization parameter that assigns the variance from $\upvarepsilon$ and has a positive value. The following restricted optimization issue is converted to a dual function using Lagrange multipliers, yielding the following final solution^20^:

7 $$f\left(x\right)=\sum_{j=1}^{n}\left({a}_{k}-{a}_{k}^{*}\right)K\left({x}_{k},{x}_{l}\right)+b$$

$K({x}_{k},{x}_{l})$ is the kernel function, ${a}_{k}$ and ${a}_{k}^{*}$ are the Lagrange multipliers with the 0k and kC restrictions, accordingly.

### Recurrent neural network (RNN)

**Figure 3:**
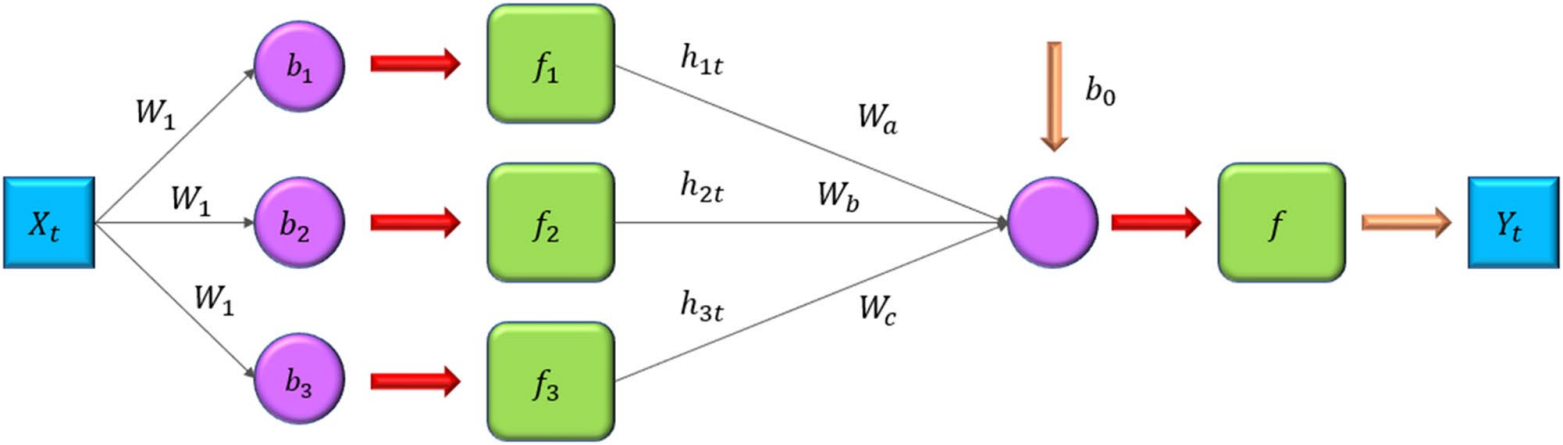
Schematic representation of the simple ANN with one hidden layer.

Deep learning approaches outperformed statistical types of ML methods, mainly when modeling non-linear situations. There are several forms of deep learning systems, with RNN being one of the most used^35^. The main difference between a basic RNN and a simple ANN approach is that the former permits hidden layer output to be utilized as an input. Figure 3 depicts a basic ANN approach with a hidden layer.

**Figure 4:**
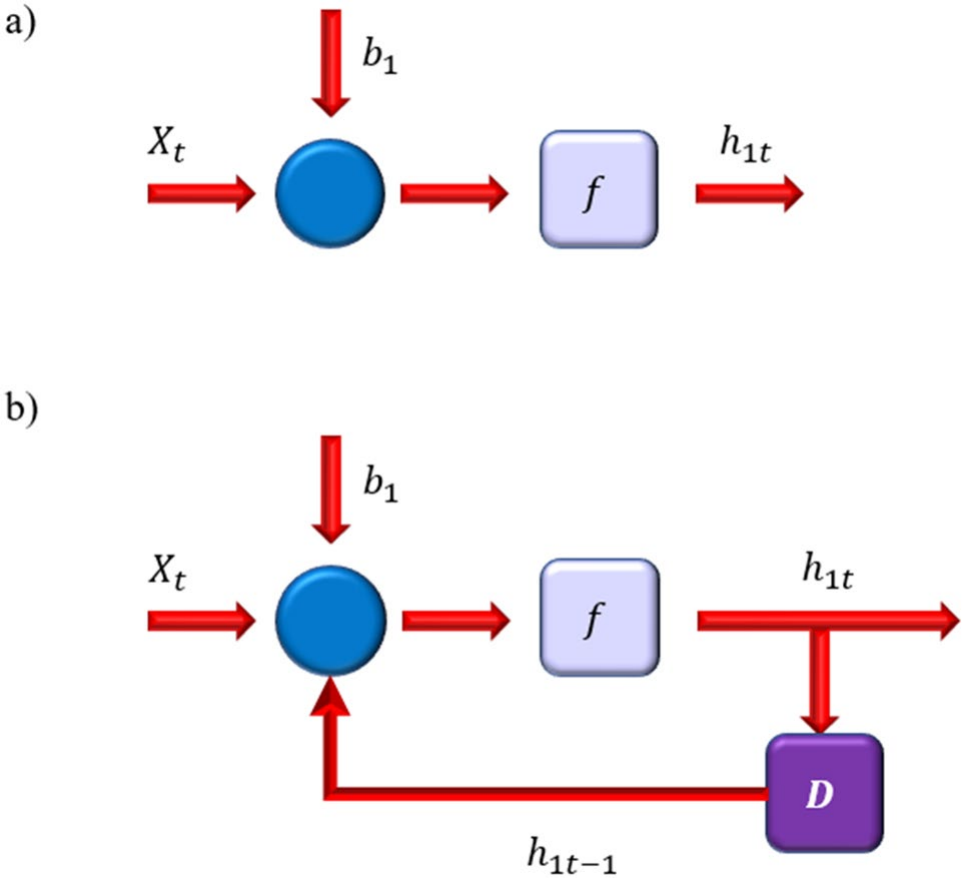
Connections around a hidden layer node in (**a**) ANN and (**b**) RNN.

Figure 4 also depicts a typical node of the hidden layer in the ANN and RNN models. The hidden layer nodes in the RNN and ANN systems behave differently, as shown in Fig. 4. The RNN comprises a unit delay (D) via which the hidden layer's output is delivered back to the neurons.

The followings are the RNN model's fundamental equations^35^:

8 $${h}_{1t}={f}_{1}({w}_{1}\times {X}_{t}+wo \times {h}_{t-1}+b1)$$

9 $${Y}_{t}={f}_{o}({w}_{a}\times {h}_{1t}+wb \times {h}_{2t}+wx\times {h}_{3t}+{b}_{o})$$

${h}_{1t}$ varies as a function of ${h}_{t-1}$ and ${X}_{t}$, as shown in Eqs. (8) and (9).

h_t: represents the hidden state at time step t. It captures the information from the previous time step and influences the current prediction. h_1t: denotes the hidden state at time step t in the first recurrent layer. RNNs can have multiple recurrent layers, and this variable refers to the first layer's hidden state. X_t: refers to the input at time step t. It could be a single data point or a sequence of data points, depending on the nature of the RNN application.

### Restricted Boltzmann machine (RBM)

**Figure 5:**
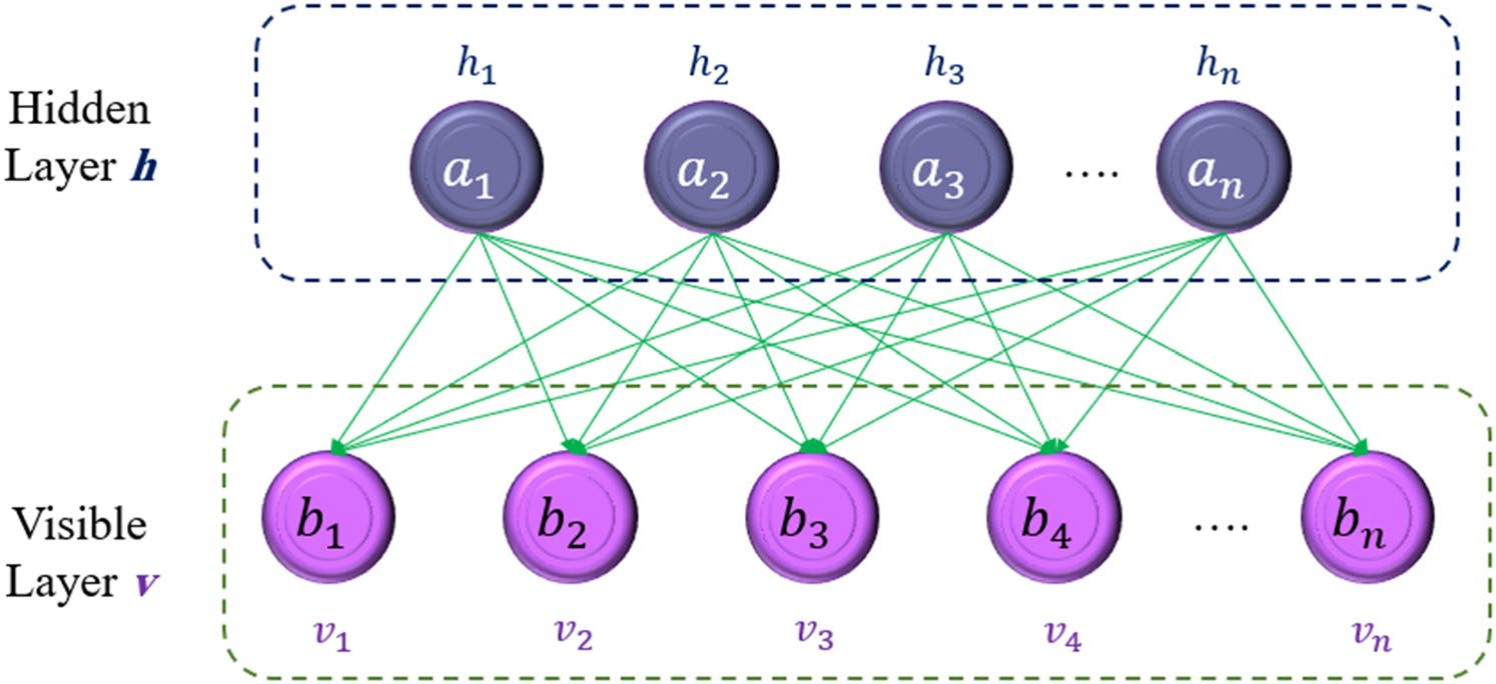
Schematic illustration of RBM model.

The RBM is one of the several types of Boltzmann machines. The RBM architecture is depicted in Fig. 5. As shown, the RBM is a neural network with two layers. The visible layer is the first, while the hidden layer is the second. Each layer is made up of neurons that are represented by circles. The visible layer's circles are linked to the hidden layer via arrows, but the neurons in the same layer are not. The rules controlling the RBM model's inner neurons, on the other hand, are predicated on the bipartite graph. The RBM method is predicated on the training of a statistical distribution between several inputs^36^.

A predetermined energy function (given in Eq. (10)) may be used to establish the probability distribution, which should be reduced to achieve the proper value of the hyperparameters^36^.

10 $$E\left(v,h\right)=-{\sum }_{i=1}^{{n}_{v}}{a}_{i}{v}_{i}-{\sum }_{i=1}^{{n}_{h}}{b}_{j}{h}_{j}-{\sum }_{i=1}^{{n}_{v}}{\sum }_{i=1}^{{n}_{h}}{h}_{i}{W}_{j,i}{v}_{i}$$

Consequently, the activation of neurons in the hidden layer is independent of the visible layer's activation and vice versa. Equation (11) expresses the conditional probability of exposed neurons in terms of the order of hidden nodes^36^.

11 $$P\left(v|h\right)={\prod }_{i=1}^{{n}_{v}}P\left({v}_{i}|h\right)$$

The activation probability as Eq. (12) may be used to generate the jth hidden layer neuron. By using the activation probability of Eq. (13), the hidden layer could also reach the ith visible neuron^36^.

12 $$P\left({h}_{j}=1|v\right)=\sigma \left({b}_{j}+{\sum }_{i=1}^{{n}_{v}}{\omega }_{ij}{v}_{i}\right)$$

13 $$P\left({v}_{i}=1|h\right)=\sigma \left({a}_{i}+{\sum }_{i=1}^{{n}_{h}}{\omega }_{ij}{h}_{j}\right)$$

As a result, the RBM requires the determination of three variables: *W*, *a*, and b^36^.

h: represents the hidden units in the RBM. These units capture latent features and interact with the visible units to learn patterns in the data. v: refers to the visible units in the RBM. These units are directly connected to the data and represent the observed variables. a, b: these are bias terms for the visible (a) and hidden (b) units. They allow the model to shift the activation threshold for the units. W: denotes the weight matrix that defines the connections and strengths between visible and hidden units. It plays a crucial role in learning the underlying data distribution.

### Deep belief network (DBN)

**Figure 6:**
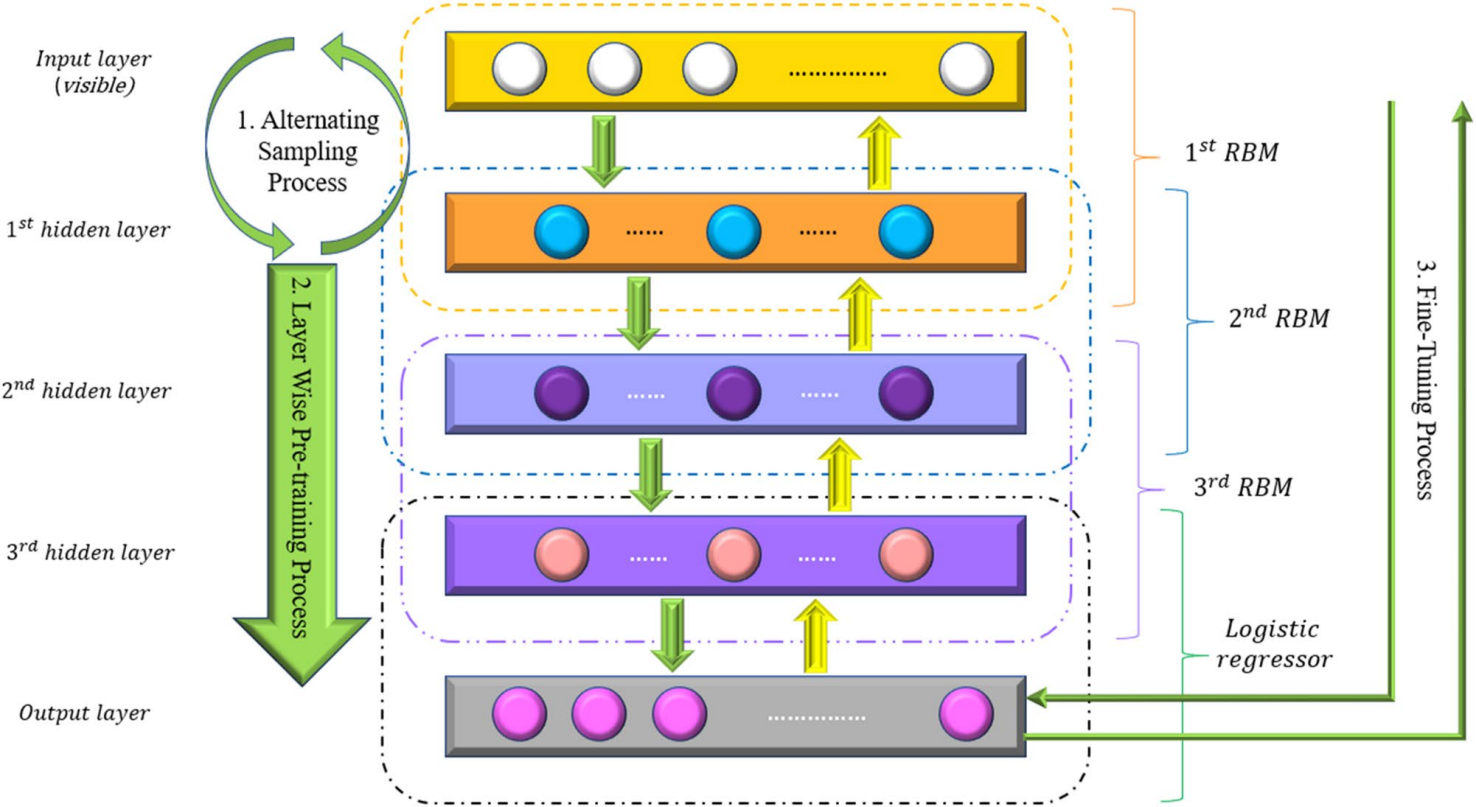
Schematic flowchart of DBN model.

Hinton^37^ was the first to suggest the DBN approach. This method is a graphical model that may be applied to various applications, including classification, image recognition, and collaborative filtering^38^. The DBN model comprises numerous layers of RBMs, each of which can be used as the visible layer for the following one. These layers provide the function of a feature extractor. Finally, the model's forecasts are created in a logistic regression layer. Figure 6 depicts a schematic representation of the DBN model. The lines in this diagram indicate the 1-alternating sampling, 2-layer-wise pre-training, and 3-fine-tuning processes, accordingly. An asymmetric weight matrix connects the first RBM's exhibited visible layer with a concealed layer. The neurons in the first hidden layer are separate from the visible neurons. As a result, sampling a vector containing hidden neurons from a subsequent distribution (*P(v|h)*) and (*P(h|v)*) is easy. As a result, the alternating sampling procedure produces a learning feature that indicates the discrepancy between the seemed neurons in the visible layer and the first hidden layer at the start and conclusion of the sampling phase^39^.

To find intrinsic characteristics in the input data and to train the first RBM, the alternation sampling procedure is used. After training the first RBM, the output from the first hidden layer is used as the input for the second hidden layer. As a result, these two hidden layers are referred to as the second RBM, and they may be pre-trained using the alternating sampling method. The greedy layer-wise pre-training method is used to learn the nth hidden layer as a new RBM using this progressive training technique. Overfitting may be avoided by using a greedy layer-wise pre-training method and alternate sampling^40^. Finally, utilizing the fine-tuning method, the settings of the entire DBN are somewhat optimized.

## Model development

### Data assembling

The aim of this study includes assaying the ability of different robust ML methods for simulating an industrial-scale of the olefin plant. For this purpose, the actual operating data of JPC, the 1184 data sets collected during successive four years, was utilized for developing DBN, RNN, MLP, and AdaBoost-SVR models. In this regard, the number of liquid and gas furnaces in service, coil outlet temperature of different furnaces, and mass flow rates of different feedstock were considered independent variables. These 24 independent variables were implemented by different ML models as input data to estimate the flow rates of ethylene, propylene, and PYG as the output of the models. During 1184 days, the mean value of each 24 parameters (i.e., input variables) was recorded every day. The statistical information of the dataset gathered in this work is tabulated in Table 2. More details about the actual operating data are provided in Supplementary Information. Python is used for the implementation of different models. The training procedure of the models was carried out using 85% of the dataset, denoted as the train subset, and the ability of the developed model was assessed using the remaining 15% of the dataset, called the test subset.

**Table 2:** Statistical details of the dataset gathered in this paper.

		Mean	STD	Min	Max
Coil outlet temperature (COT) for each furnace	Liquid furnaces (NO.)	2.98	0.48	0.79	3.94
	Gas furnaces (NO.)	4.51	0.65	1.31	5.82
	F-101 (°C)	786.39	190.88	21	853
	F-102 (°C)	790.74	179.14	30	937
	F-103 (°C)	775.23	210.72	26	847
	F-104 (°C)	770.05	212.88	25.7	858.3
	F-105 (°C)	779.29	199.45	20	853
	F-106 (°C)	749.59	246.97	20.23	857
	F-107 (°C)	764.72	220.31	17.44	861
	F-108 (°C)	734.33	258.49	28	859.2
	F-109 (°C)	802.84	156.96	22.89	853.3
	F-110 (°C)	734.09	264.05	22.27	862
Flow rate of feed streams	Fresh ethane 1 (ton/day)	622.84	373.31	0	1558.14
	Fresh ethane 2 (ton/day)	1917.24	434.52	512.59	3099.41
	C5 Cut (ton/day)	152.01	55.93	0	312.01
	Light ends (ton/day)	2405.38	532.37	65.09	3619.68
	LPG (ton/day)	169.91	114.22	0	428.67
	C5+ feed (ton/day)	99.16	118.18	0	372.03
	C9+ cut (ton/day)	73.09	25.33	4.12	169.93
	C3+ cut (ton/day)	311.47	131.67	0	728.25
Flow rate of recycle streams	Ethylene (ton/day)	48.62	15.31	0	100.43
	Ethane (ton/day)	432.01	166.39	0	1382.49
	Butane (ton/day)	256.43	346.94	0	1560.05
	C4 cut recycle (ton/day)	335.13	129.91	0	696
Flow rate of product streams	Ethylene	3122.55	388.61	575.76	3703.11
	Propylene	761.75	118.66	12.45	1382.37
	Pyrolysis gasoline	779.71	121.68	157.36	1054.97

In this study, the optimization of hyperparameters for the models was conducted through the implementation of grid search on the dataset. The selection of hyperparameters was carefully considered to enhance the model's performance. Additionally, to prevent potential overfitting issues during the training phase, a rigorous approach was adopted, involving the utilization of a sixfold cross-validation technique. This enabled the dataset to be divided into six subsets, allowing each subset to serve as both training and validation data iteratively. By employing this well-established validation method, the models' generalization capabilities were effectively assessed, enhancing the reliability of the results obtained.

Each model's grid search hyperparameters were different, and the importance of the hyperparameters was determined by theoretical and practical considerations. For each model, the following hyperparameters were employed. Table 3 shows the optimal values of the major hyperparameters, as well as the search intervals for the hyperparameters set for the machine learning models used in this study.

**Table 3:** Optimal features for implemented models.

Model	Hyperparameter	Search range	Optimum value/feature
DBN	DBN network structures	1–25	[5, 10, 15, 20, 15]
	Learning rate	0.9–0.0001	0.25
	Maximum epochs of RBM	1–500	100
		0.01–0.9	0.35
MLP	Number of hidden layers	1–5	1
	Neurons' structure in hidden layer	–	[17]
	Learning role	–	Bayesian regularization
Adaboost-SVR	n_estimators	1–200	50
	Learing_rate	0.01–0.9	0.15
	Kernel type	Linear, polynomial, radial basis function	Polynomial/radial basis function
	Regularization parameters C	1, 1e1, 1e2..1e5	1
	Degree for polynomial function	1–4	2
RNN	Number of epochs	1–50	25
	Learing_rate	0.01–0.9	0.02
	Batch size	1–100	50
	Model of RNN	–	Rnn

### Detection of the outliers

The reliability and accuracy of the models are highly dependent on the applicability of the actual employed data. Always some imprecise measurements can be found in the recorded data points, which can drastically affect the accuracy of the developed models as a threat. Therefore, to develop a reliable model, these outliers should be detected and eliminated from the recorded data set. In this study, the Leverage method^41–43^ was used for finding outliers, and the Hat matrix of the Leverage method was constructed as Eq. (14)^20, 44^:

14 $$H=X{({X}^{T}X)}^{-1}{X}^{T}$$

In Eq. (14), X is a matrix with dimensions of p $\times$ q, where p is the number of data points, and q is the number of independent variables. The diagonal elements of the Hat matrix denote the hat value of data. Outliers can be identified by William’s plot that the normalized residuals are illustrated with respect to the hat value. The warning Leverage value $\left({H}^{*}\right),$ which can be calculated by Eq. (15), is also shown on William’s plot^19, 41–43^.

15 $${H}^{*}=\frac{3(q+1)}{p}$$

### Assessment of the accuracy

The capability of the developed models is assessed using different statistical techniques, which are described bellows^45, 46^:

The average absolute error between the model-predicted and the target value is calculated by the average absolute percent relative error (AAPRE) using Eq. (16):

16 $$AAPRE\left(\%\right)=\frac{100}{n}\sum_{i=1}^{n}\left|\frac{O{(i)}_{Pred}-O{(i)}_{Exp}}{O{(i)}_{Exp}}\right|$$

The root mean square error (RMSE) was used to determine the error dispersion. Equation (17) shows the mathematical equation of RMSE:

17 $$RMSE={\left(\frac{{\sum }_{i=1}^{n}O{\left(i\right)}_{Pred}-O{(i)}_{Exp}{)}^{2}}{n}\right)}^{1/2}$$

The data dispersion is indicated by Standard deviation error (STD) using Eq. (18). The lower the STD value, the lower is data dispersion.

18 $$STD= \left(\frac{1}{n-1}\sum_{i=1}^{n}{\left(\frac{O{(i)}_{Pred}-O{(i)}_{Exp}}{O{(i)}_{Exp}}\right)}^{2}\right)^{1/2}$$

Another parameter for assessment of the model accuracy is the coefficient of determination $\left({R}^{2}\right)$. The closer to 1, the better predicting experimental data by ${R}^{2}$ value. The ${R}^{2}$ value can be calculated using Eq. (19):

19 $${R}^{2}=1-\frac{{\sum }_{i=1}^{n}{\left(O{(i)}_{Pred}-O{(i)}_{Exp}\right)}^{2}}{{\sum }_{i=1}^{n}{\left(O{(i)}_{Exp}-{\stackrel{-}{O(i)}}_{Exp}\right)}^{2}}$$

In Eqs. (16–19), O is the flow rates of the produced PYG, propylene, and ethylene. n depicts the number of industrial data.

## Results and discussion

### Model assessment

**Figure 7:**
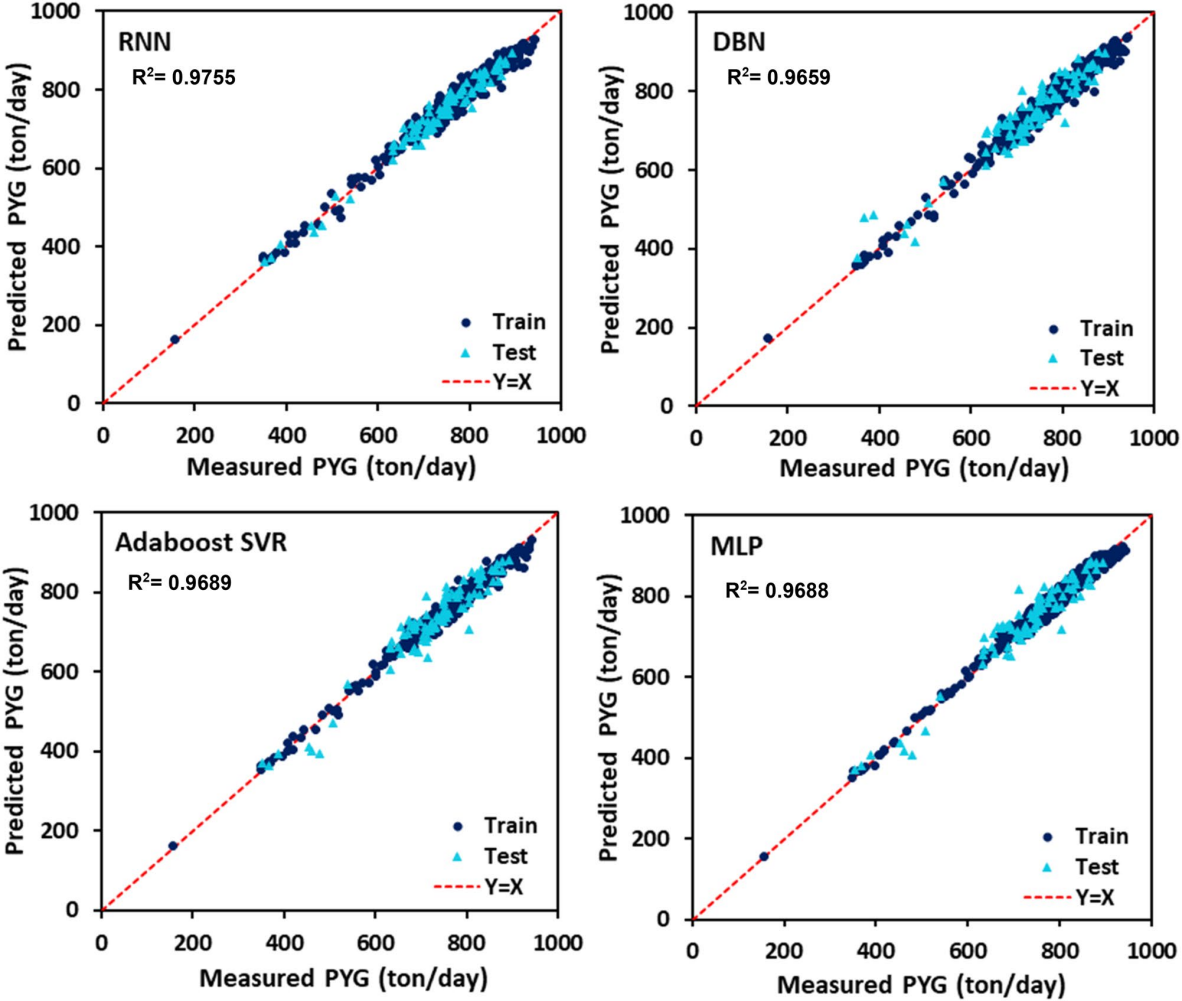
Crossplots of the proposed ML models for PYG in this study.

**Figure 8:**
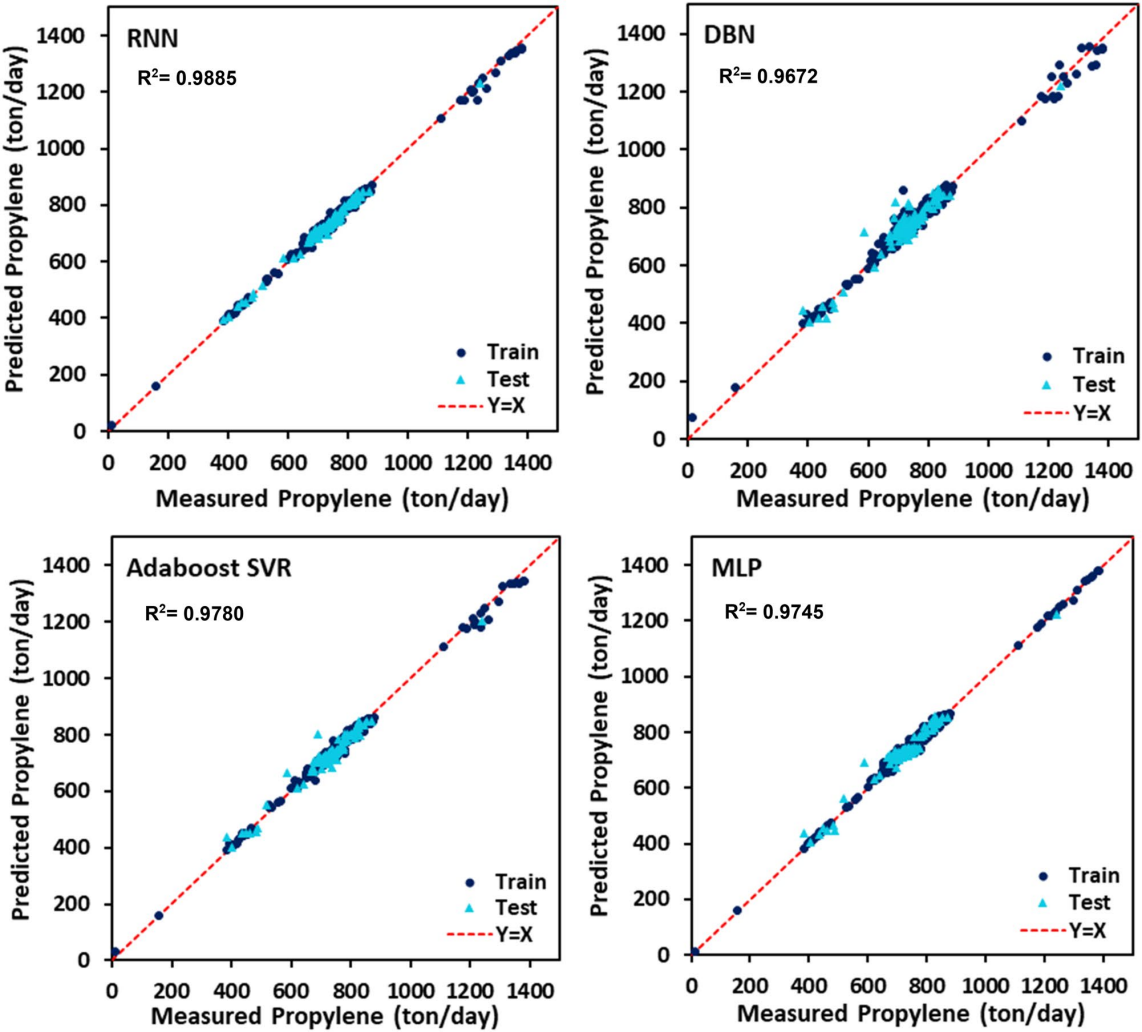
Crossplots of the proposed ML models for propylene in this study.

**Figure 9:**
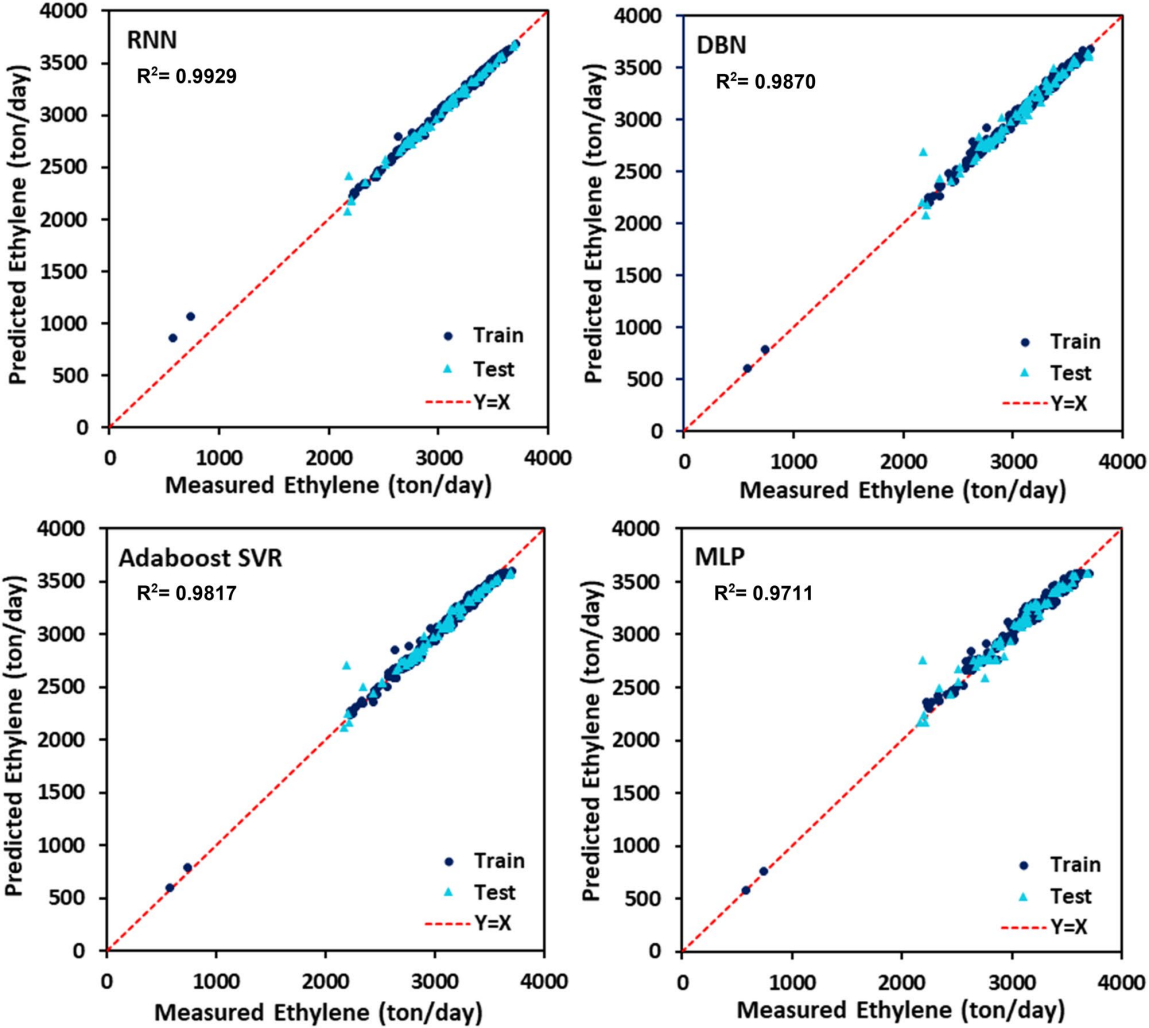
Crossplots of the proposed ML models for ethylene in this study.

The PYG, propylene, and ethylene flow rates estimated by DBN, RNN, MLP, and AdaBoost-SVR models with respect to the experimental data are plotted in Figs. 7, 8 and 9. It can be seen that all of the developed models exhibited outstanding performance, and the data points were dispersed very closely to the line Y = X. For a more accurate assessment, the statistical parameters belonging to the training, testing, and total data set of each model attributed to the predicted values of PYG, propylene, and ethylene are listed in Tables 4, 5 and 6. It can be seen that for all three products, the RNN model indicated the best performance with the highest ${R}^{2}$ and the least AAPRE, RMSE, and STD for the total data set. In addition, the RNN model exhibited excellent performance in the training and testing stages. Based on Tables 4, 5 and 6, the best accuracy obtained from the RNN model (i.e., total AAPRE of 0.7%) is related to ethylene. The minimum accuracy is assigned to PYG with a total AAPRE of 1.9%. However, these low levels of AAPRE confirmed that the developed RNN model in this work was a reliable model for predicting the flow rates of the main products on the industrial scale of the olefin unit.

**Table 4:** Calculated statistical criteria for the developed models for PYG.

Statistical criteria		AAPRE %	R^2^	STD	RMSE
RNN	Train	1.907	0.9757	0.0249	18.942
	Test	2.089	0.9745	0.0264	19.359
	Total	1.944	0.9755	0.0253	19.026
DBN	Train	1.915	0.9779	0.0245	18.151
	Test	3.771	0.9188	0.0517	34.598
	Total	2.287	0.9659	0.0322	22.430
MLP	Train	1.687	0.9829	0.0205	15.890
	Test	3.798	0.9122	0.0499	35.980
	Total	2.111	0.9688	0.0289	21.473
AdaBoost-SVR	Train	1.592	0.9813	0.0204	16.613
	Test	3.696	0.9192	0.0489	34.492
	Total	2.014	0.9689	0.0285	21.422

**Table 5:** Calculated statistical criteria for the developed models for propylene.

Statistical criteria		AAPRE %	R^2^	STD	RMSE
RNN	Train	1.288	0.9891	0.0221	12.809
	Test	1.281	0.9847	0.0161	12.330
	Total	1.286	0.9885	0.0209	12.714
DBN	Train	3.059	0.9031	0.0508	31.395
	Test	2.339	0.9781	0.1668	18.164
	Total	2.483	0.9672	0.1509	21.475
MLP	Train	1.624	0.9833	0.0210	15.813
	Test	2.734	0.9207	0.0450	28.126
	Total	1.846	0.9745	0.0276	18.930
AdaBoost-SVR	Train	1.604	0.9859	0.0541	14.557
	Test	2.511	0.9306	0.0416	26.321
	Total	1.785	0.9780	0.0519	17.555

**Table 6:** Calculated statistical criteria for the developed models for ethylene.

Statistical criteria		AAPRE %	R^2^	STD	RMSE
RNN	Train	0.722	0.9933	0.0263	32.489
	Test	0.638	0.9918	0.0150	32.812
	Total	0.705	0.9929	0.0245	32.554
DBN	Train	0.727	0.9947	0.0101	28.640
	Test	1.519	0.9475	0.0372	80.628
	Total	0.886	0.9870	0.0189	44.242
MLP	Train	1.354	0.9817	0.0178	53.580
	Test	1.942	0.9172	0.0467	99.274
	Total	1.472	0.9711	0.0263	65.948
AdaBoost-SVR	Train	1.001	0.9902	0.0120	39.157
	Test	1.625	0.9383	0.0407	87.432
	Total	1.126	0.9817	0.0212	52.503

**Figure 10:**
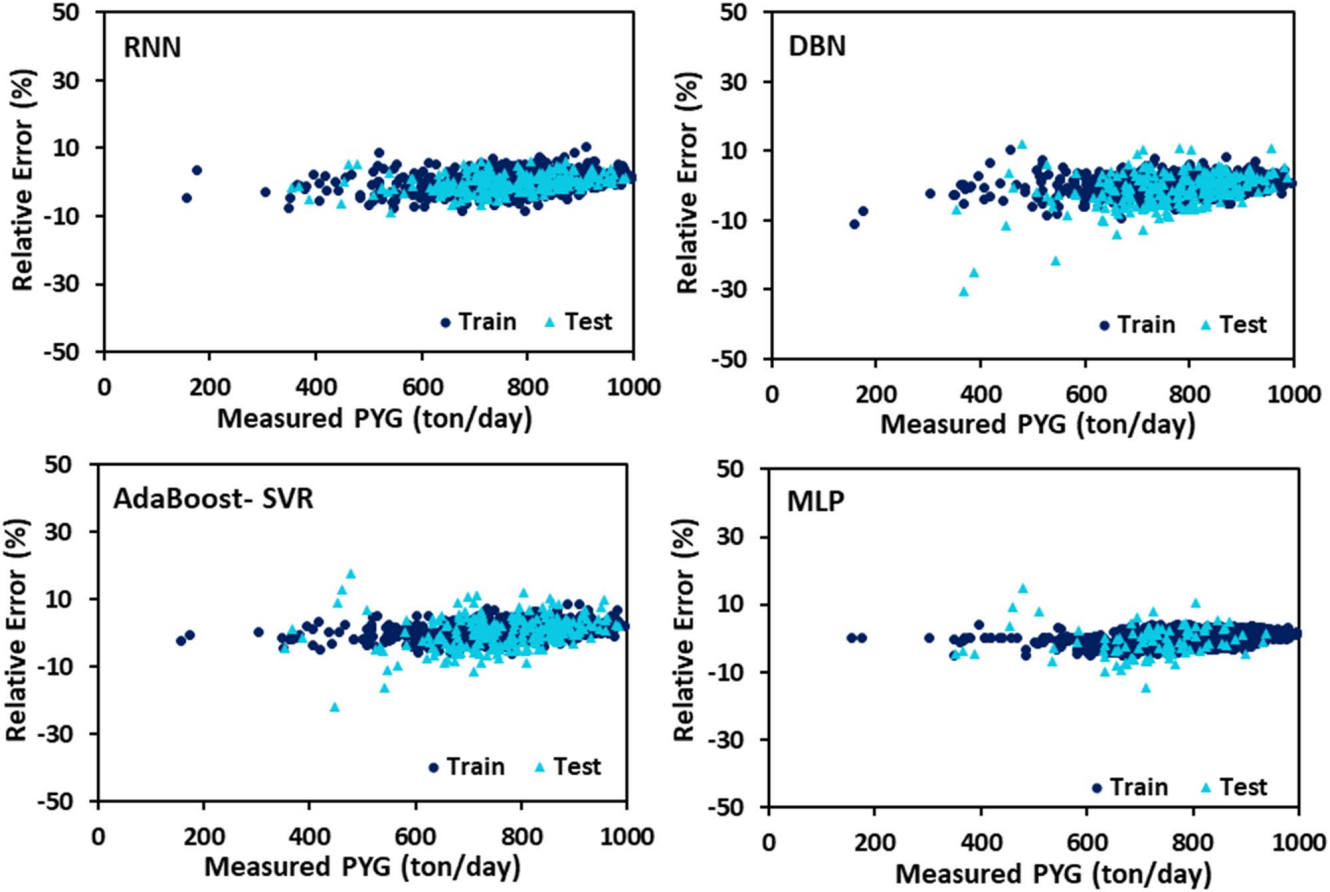
Error distribution plots of ML models for PYG training and test sets.

**Figure 11:**
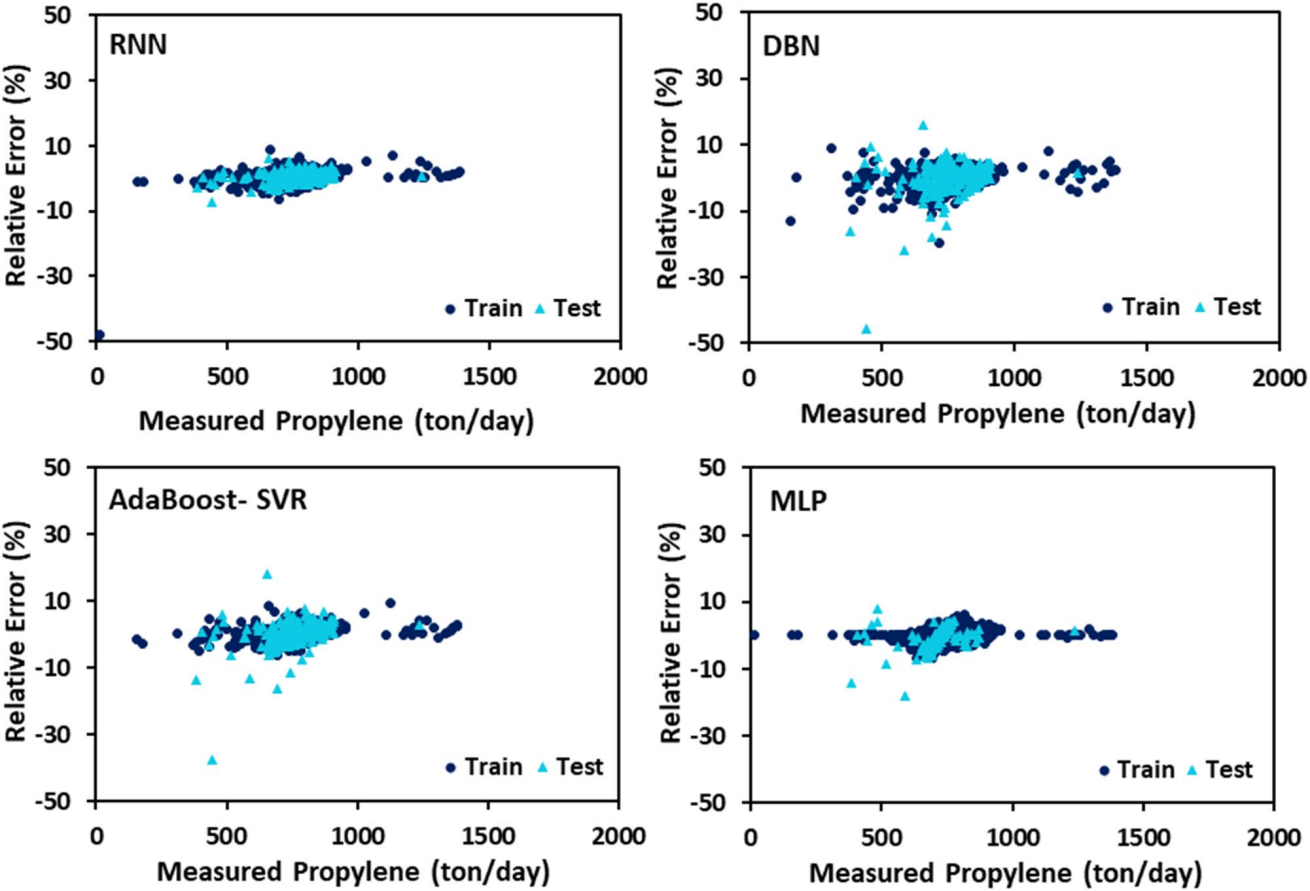
Error distribution plots of ML models for propylene training and test sets.

**Figure 12:**
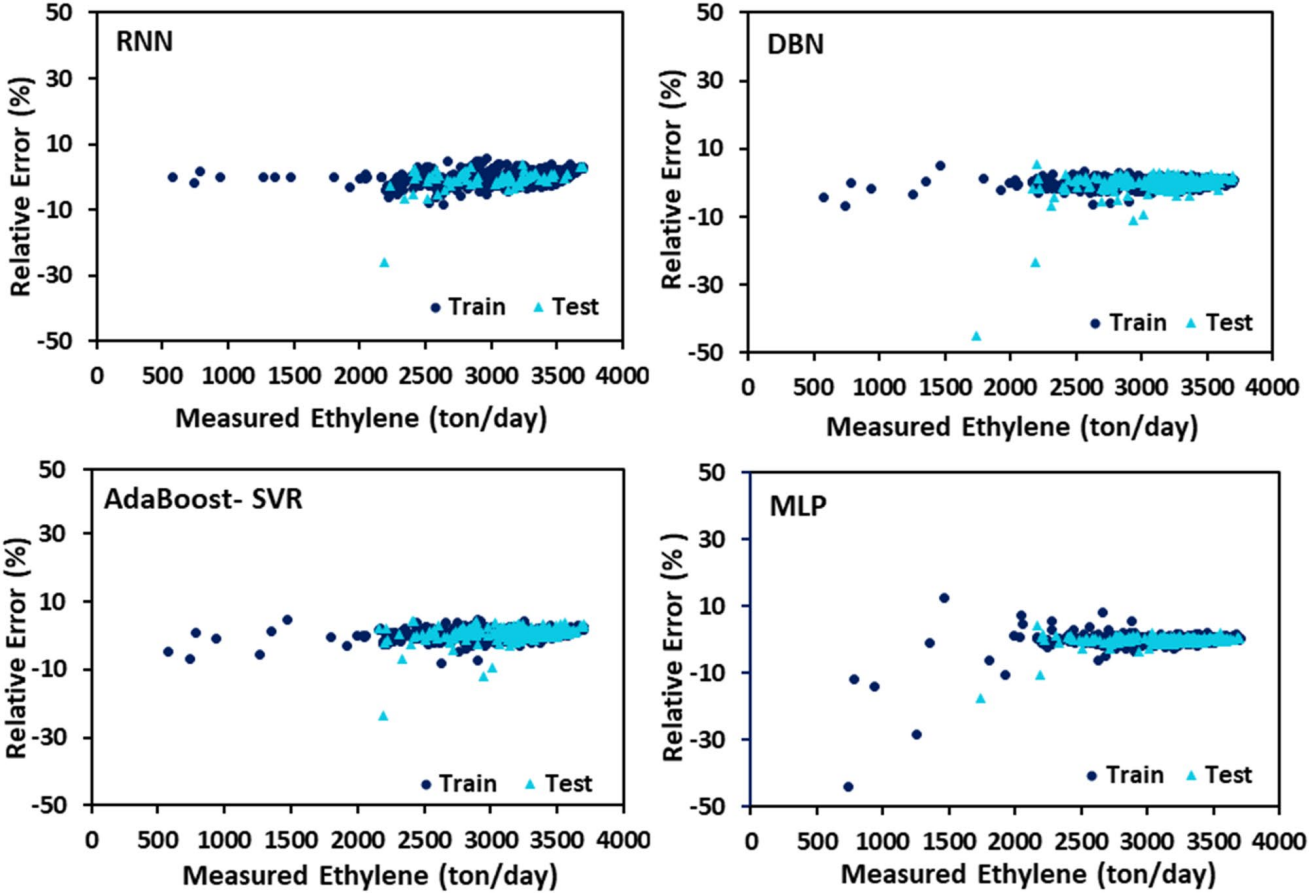
Error distribution plots of ML models for ethylene training and test sets.

**Figure 13:**
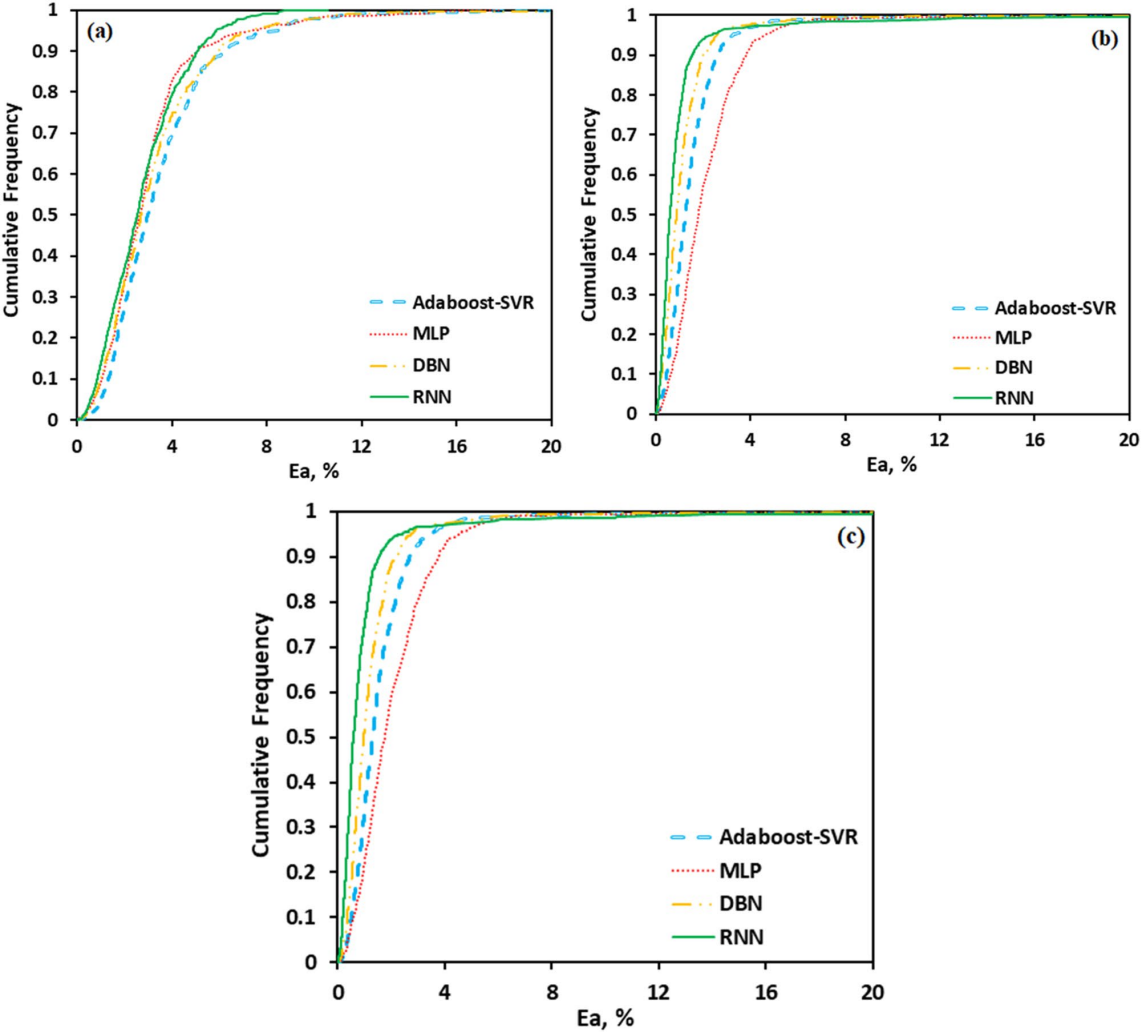
The cumulative frequency plot for the developed predictive models: (**a**) PYG, (**b**) propylene, and (**c**) ethylene.

Error distribution for both train and test data sets obtained by different developed models is plotted in Figs. 10, 11 and 12. Error fluctuations on both sides of the zero line indicate that all models have provided acceptable accuracy for all three main products. The slight deviations of the RNN model from the zero line indicated the greater accuracy of this model, especially for PYG and propylene estimation. The cumulative frequency of data points regarding the absolute relative error is illustrated in Fig. 13. In these diagrams, any model closer to the vertical axis has higher accuracy in the prediction of experimental data. Based on Fig. 13, for ethylene prediction using the RNN model, approximately 85% of data points have a relative error smaller than 2%. While for example, when the MLP model was employed, only less than 25% of the data predicted for ethylene had a relative error lower than 2%. Accordingly, the RNN model presented higher accuracy with respect to the other models for predicting PYG and propylene.

### Applicability of the range of RNN model

**Figure 14:**
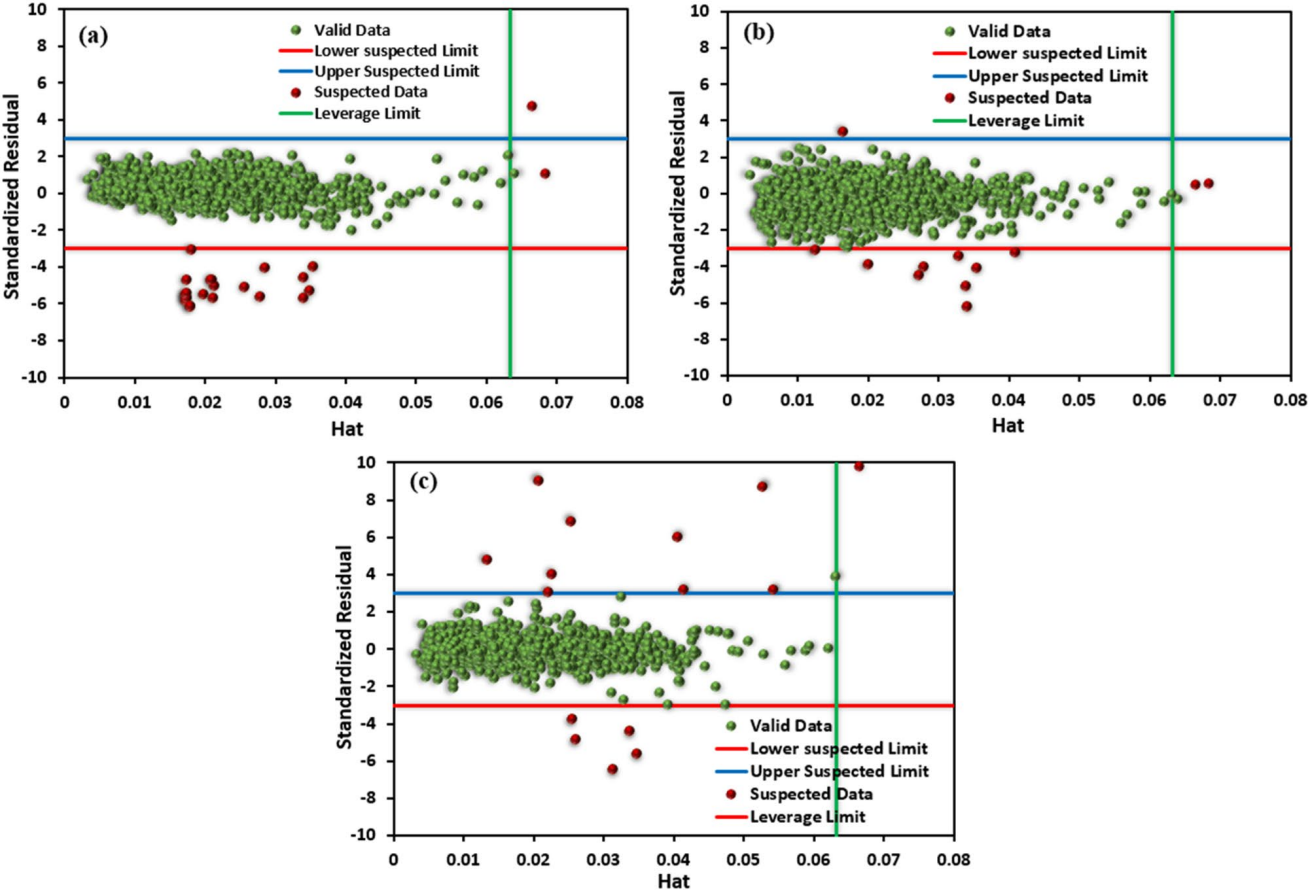
Williams plot for outlier the proposed RNN model for (**a**) PYG, (**b**) propylene, and (**c**) ethylene.

To evaluate the applicability domain of the developed RNN model, William’s plots for PYG, propylene, and ethylene are illustrated in Fig. 14. Based on the Leverage method^41–43^, the cut-off values are recommended as $\pm 3$ of the normalized residuals on the y-axis and 0 to ${H}^{*}$ on the x-axis. The data dispersed within these boundaries are considered valid data^47^. It can be seen in Fig. 14 that for all three main products, the majority of data points existed within the acceptable domain, indicating that the developed RNN model was statistically accurate and reliable. However, a few points of the data set were out of the specified range; accordingly, these points were considered doubtful data. In this study, an industrial-scale olefin plant was simulated over four years. The existence of some outliers is inevitable. Some of them are indeed related to the complexity of the plant. In addition, some inaccurate data may have been recorded during unexpected situations. Since the suspected data was insignificant compared to the entire data set, it can be concluded that both the actual data of the plant and the developed RNN model were accurate and reliable.

### Sensitivity analysis

The sensitivity analysis was performed to evaluate the extent of the effects of all input variables on the PYG, propylene, and ethylene flow rates predicted using the RNN model. The measure of r (relevancy factor) determines the magnitude of the effect of each input variable on the main products flow rates as the model outputs^48^. r can be negative or positive. The positive value of r for one input variable indicates the direct interaction between output and that input parameter. On the other hand, the negative r confirms the inverse relationship^49^. The greater the absolute value of r for an input variable, the more significant the impact of that parameter on the model output^50^. Relevancy factor (r) is calculated using Eq. (20)^19^:

20 $$r\left({I}_{i},\omega \right)=\frac{{\sum }_{j=1}^{n}\left({I}_{ij}-\overline{{I }_{i}}\right)\left({\omega }_{j}-\overline{\omega }\right)}{{\left({\sum }_{j=1}^{n}{\left({I}_{ij}-\overline{{I }_{j}}\right)}^{2}{\sum }_{j=1}^{n}{\left({\omega }_{j}-\overline{\omega }\right)}^{2}\right)}^{0.5}}$$

where ${\omega }_{j}$ and $\overline{\omega }$ represent the jth and the mean value of the predicted products flow rate, respectively. ${I}_{ij}$ and $\overline{{I }_{i}}$ denote the jth and the mean value of the i-th input parameters, respectively. n is the number of data sets.

**Figure 15:**
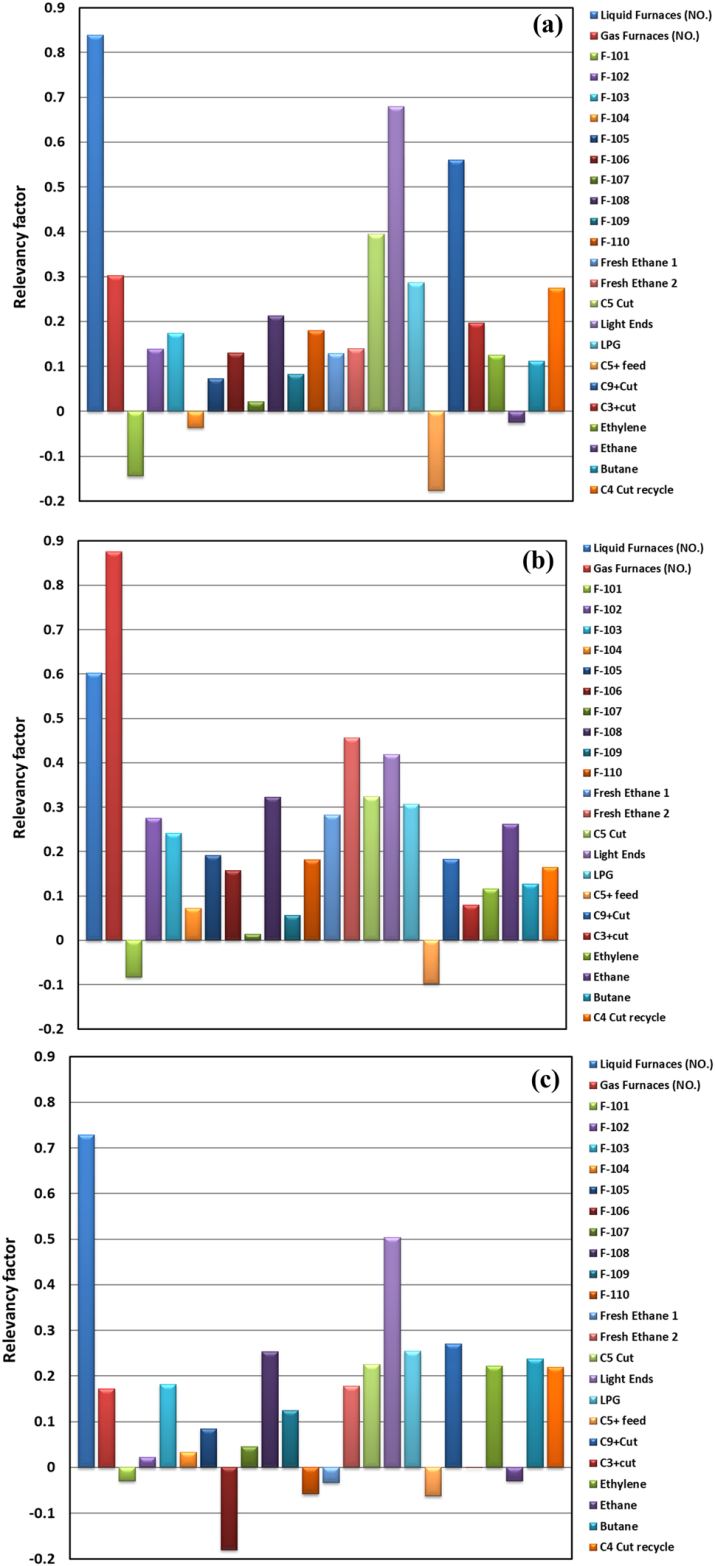
Sensitivity analysis for RNN approach based on different influential parameters: (**a**) PYG, (**b**) ethylene, and (**c**) propylene.

The calculated relative importance of all the inputs on the PYG, propylene, and ethylene flow rates based on the RNN model is shown in Fig. 15. As mentioned earlier, the input variables were the flow rates of different feedstock, numbers of active furnaces, and coil outlet temperature of furnaces. Among them, the numbers of operational furnaces and flow rates of some feedstock, especially Light ends, affected more significantly the main product flow rates. While coil outlet temperature represented negligible effects on the model outputs. As seen in Fig. 15, the number of liquid furnaces in service, as well as the flow rates of Light ends and C_9+_ cut, were input variables that exhibited the most positive effect on the produced PYG flow rate. This means that any increase in these special inputs would increase the amount of PYG. For propylene flow rate, the inputs with the most positive effects were also the amount of active liquid furnaces and Light ends flow rate. At the same time, ethylene flow rate was highly influenced by the number of gas furnaces. Whereas the temperature of the F-101 furnace and C_5+_ flow rate inversely affected all three products’ flow rates, their influences were minor. Therefore, as the amount of these particular inputs increases, the amount of PYG, ethylene, and propylene decreases slightly. The propylene flow rate decreased a little by increasing the temperature of the F-106 furnace.

### Effects of the operating conditions on the cracking severity factor

Understanding the effects of different operating conditions on the cracking process is important to ensure optimum reaction conditions. In the cracking processes, cracking severity factors characterize the operating conditions to maximize main product yields and minimize operational costs^51^. The cracking severity factors determine the extent of the breaking down of the feed molecules and the distribution of the products. These factors, as independent indices from the reactor scale, allow one to investigate the effects of different operating conditions and maintain the desired product distribution. There are various practical definitions for cracking severity indices^52^. One of the most currently used indices is the propylene to ethylene (P/E) ratio^51^. In industrial practices, the target value of the P/E ratio is around 0.55 to avoid over- and under-cracking^53^. Higher values of P/E ratios indicate the under-cracking and production of high amounts of liquid by-products. On the other hand, lower values of P/E ratios correlate with the increased production of coke.

**Figure 16:**
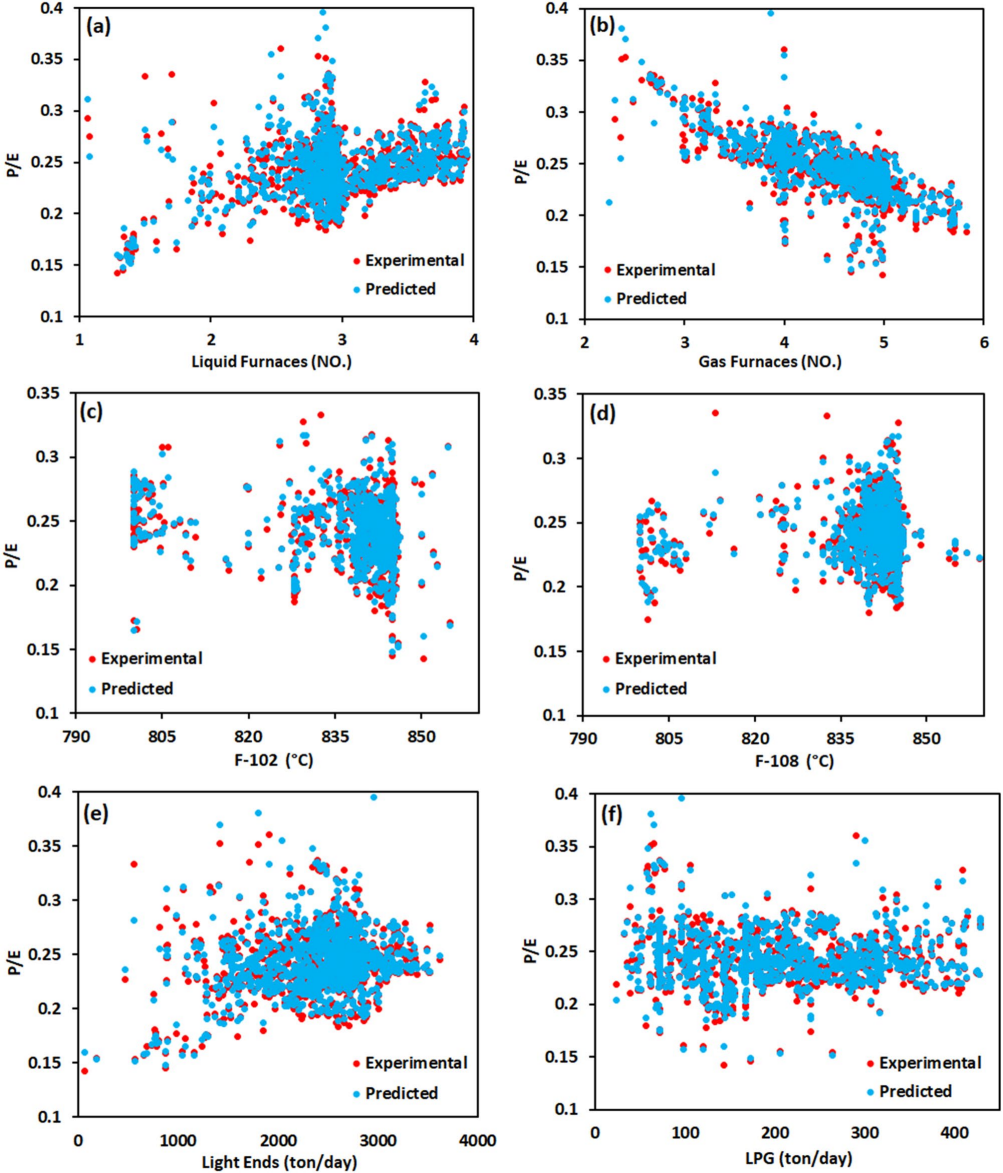
The effect of input parameters on the ratio of propylene/ethylene (P/E).

In this study, the predicted P/E ratios using the RNN model at different operating conditions are compared with experimental values in Fig. 16. The operating conditions were the number of active liquid and gas furnaces, the outlet temperature of F-101 and F-108 furnaces, and the flow rates of Light ends and LPG selected as the most effective operating parameters based on the sensitivity analysis. As shown in Fig. 16, the experimental values of P/E ratios dispersed in the range of 0.15–0.35 in different conditions, resulting in high coke formation. However, the developed RNN model predicted the experimental P/E ratios with high accuracy at different operating conditions. As shown in Fig. 16a, b, the P/E ratios gradually increased by increasing the number of operational liquid furnaces. While increasing the number of active gas furnaces favored the ethylene yield. The feed composition in the cracking processes is one of the crucial factors affecting product distribution and has been highly investigated by different researchers^54, 55^. Light olefins are produced mainly from alkanes, the higher amounts of n-alkanes result in a higher ethylene yield. By changing the feedstock from alkanes to liquid compounds, cracking has not occurred much, and large quantities of liquid products such as PYG are produced.

The effects of feed composition on the P/E ratios are also illustrated in Fig. 16e at different Light ends flow rates. From Fig. 16e, P/E ratios increase slightly with elevating Light ends flow rates. This is also primarily due to the higher concentration of liquid feedstock, causing under-cracking and producing more liquid products^52^. As seen in Fig. 16f, LPG flow rates exhibited negligible effects on the P/E ratios. This observation confirmed that referring solely to the feedstock composition is not sufficient for controlling the P/E ratios. To keep the process favorable towards the light olefin production, other important factors such as the temperature of furnaces need to be justified. The effects of the coil outlet temperature of F-102 and F-108 furnaces on the P/E ratios are indicated in Fig. 16c, d. The temperature of these furnaces was not changed much and was kept in the range of 835–850 °C. Since cracking reactions have endothermic nature, the temperature of furnaces required to be maintained as high as about 850 °C. Beyond the mentioned range, side-reactions are accelerated, and different by-products are formed. Higher temperature favors the coke formation inside the reactor tube, decreasing the furnace lifetime^54^. In addition, it was reported that the higher temperature causes the reaction of ethane and ethylene with other components leading to the production of more methane and other by-products^52^. At lower temperatures, the yields of secondary products increase, owning the acceleration of oligomerization reactions^56^. As a result, it is essential to employ the optimum temperature for furnaces to prevent the formation of by-products and maximize ethylene and propylene formation.

As mentioned above, the effects of operating conditions on the product distribution of cracking could be well studied by cracking severity factors. The analysis of severity factors will enable engineers to evaluate cracking performance from different points of view to ensure optimum olefin yields. This study confirmed that the severity factors can be well predicted by the ML approaches, and these models could be used as powerful and robust alternatives for expensive and time-consuming experimental investigations to control and optimizing of industrial plants.

It should be pointed out here that the proposed RNN model should be coupled with some optimization algorithms such as genetic algorithm, particle swarm optimization, etc. to optimize the real JPC plan. This could be the objective of our future research. Indeed, after finding the optimal conditions, we should perform those conditions in the real JPC plant ensuring the good performance of the optimization algorithm.

## Conclusions

In this study, the ability of four powerful ML models, namely MLP, AdaBoost-SVR, RNN, and DBN, was evaluated to predict the main products flow rates of an industrial-scale olefin plant using actual data gathered over four years. The flow rates of different feedstock, the coil outlet temperature of different furnaces, and the number of liquid and gas furnaces in service were used as input variables to forecast ethylene, propylene, and PYG flow rates. Among the developed models, the RNN was the most accurate one, which can predict the output flow rates with AAPRE of 0.7% to 1.9%. The extent of the effects of different input variables on the products was studied using the relevancy factor. In addition, the P/E ratio as a cracking severity factor was employed to investigate the effects of different operating conditions on the product distribution. This study proved that these simple and cost-effective models could be beneficially employed for studying and simulation complex and industrial-scale real plants in replacing costly and time-consuming experimental work (Supplementary Information).

### Supplementary Information

Supplementary Information.

## Data Availability

All the data are available from the corresponding author on reasonable request.
